# Oral Hygiene in a Sample of Children/Adolescents Living in Family-Homes from the Province of Milan (Italy): A Pilot Study

**DOI:** 10.3390/dj8020033

**Published:** 2020-04-09

**Authors:** Alessandro Nota, Floriana Bosco, Shideh Ehsani, Francesca Giugliano, Giulia Moreo, Simona Tecco

**Affiliations:** 1 Dental School, Vita-Salute San Raffaele University and IRCCS San Raffaele Hospital, via Olgettina 48, 20132 Milan, Italy; nota.alessandro@hsr.it (A.N.); boscofloriana@gmail.com (F.B.); shideh_ehsani@yahoo.it (S.E.); giugliano.francesca2@gmail.com (F.G.); 2 Dental School, Milano-Bicocca University, 20132 Milan, Italy; moreo.giulia@gmail.com

**Keywords:** oral hygiene, disadvantaged children, plaque index, motivational program

## Abstract

Objective: This pilot study is a prospective controlled clinical trial, designed to evaluate the short-term clinical results (the plaque index) of an educational/motivational program for home oral hygiene, directed to children and adolescents who live in family-homes. Methods: The setting of the project was the province of Milan (Italy), where two family-homes were selected. The study group included 26 children (16 females and 10 males) aged between 7 and 15 years, of Italian nationality, from the family-home communities. The control group included 26 children (15 females and 11 males, aged between 7 and 15 years) of Italian nationality, matched for age and gender distribution with the study group, that were not in a socially disadvantaged condition. Collection of the plaque index (PI) was performed at t0. Then, all basic oral hygiene instructions were given to all children/adolescents and their educators. Education and motivation were repeated in the same way after 4–7 weeks (T1), and after 10–12 weeks (T2). The PI was taken also at T1 and T2. Results: An improvement in the PI was generally found in both groups, but there was no statistically significant difference between the two groups over time. Multivariate analysis of variance (MANOVA) revealed a statistically significant effect of time [F (1, 52) = 90.73, *p* < 0.001], regardless of the assignment group, in consequence of which the plaque index presented a moderate and significant improvement. Conclusion: The present data confirm the validity of the educational/motivational program to improve oral hygiene in children/adolescents, regardless of the assignment group.

## 1. Introduction

Health is defined by the World Health Organization (WHO) as “a state of complete physical, mental and social well-being and not merely the absence of disease”. Health is thus considered a fundamental right of every individual and, for this reason, there should be no inequality with respect to access to information and services to protect it, mostly by the disadvantaged social groups [1,2].

One of the most disadvantaged social groups consists of children/adolescents that are removed from their families of origin, and are consequently institutionalized in family-homes: these minors are generally entrusted to educators/guardians that intervene to guarantee their protection, educational support, and accompaniment towards autonomy with the support of the National Health System and Social Services. However, not being able to enjoy the love and protection of their own parents, they seem to be often neglected, with serious consequences on their health [3].

Oral health is an integral part of health in general [4,5] and out of all the oral pathologies, caries and periodontal disease are the most frequent, and they can both be linked to poor oral hygiene [6].

Therefore, an adequate oral hygiene regime at home represents the best prevention strategy for these problems [7].

Some studies have demonstrated that factors such as age, race, gender, oral hygiene habits, socio-economical status, and oral hygiene education can define the state of oral hygiene, and improving the knowledge on oral hygiene can bring to a greater awareness, especially regarding children and adolescents [8,9,10,11].

Some studies in the literature suggest that socially disadvantaged children, for example, orphans and children brought up in family homes, tend to lack the correct knowledge of oral hygiene procedures. This causes an increased prevalence of caries and periodontal problems compared with other children [12,13,14].

The present study aimed to evaluate the short time clinical results (the plaque index) of an educational/motivational program to improve home oral hygiene, directed to children and adolescents who live in family-homes in the province of Milan (Italy). 

## 2. Subjects and Methods

The present study was a pilot clinical trial, prospective and controlled, that aimed to evaluate the short-term results of an educational/motivational program on home oral hygiene, directed to children and adolescents who live in family-homes. The setting of the project was the province of Milan (Italy), where two family-homes were enrolled: the communities “Tutti per uno” and “Girotondo”, belonging to the Center for Childhood Trauma and Family, managed by the cooperative “Spazio Aperto Servizi Cooperative” in the same city. These family-homes are specifically organized for boys and girls aged between three and fifteen years. Their integrated clinical–educational work with children/adolescents and with their families is mostly oriented towards focal and multimodal interventions that encourage children/adolescents to develop specific skills for discharge. The acquisition of specific skills for discharge represents the positive outcome of the work of “post-traumatic repair” for the child/adolescent and their own family. All the children/adolescents had been removed from their families of origin, following domestic abuse and maltreatments, or due to family poverty in particularly critical and delicate situations, which strongly compromised the parental relationship (most of these minors had been entrusted to the structures without the possibility of any contact with their nucleus of origin, after the parental authority had been removed).

The study was conducted in accordance with the Declaration of Helsinki, and the protocol was approved by the Ethics Committee of the San Raffaele Hospital (Project VARIA2, 23 June 2016).

The study group included 26 children (16 females and 10 males) aged between 7 and 15 years (mean age: 11.71 ± 2.08), of Italian nationality.

The control group included 26 children, 15 females and 11 males, aged between 7 and 15 years (mean age: 10.63 ± 1.86), of Italian nationality, matched for age and gender distribution with the study group, all relating to the National Health System (S.S.N.) and private practice of the Department of Dentistry of the San Raffaele Hospital in Milan (Italy), that were not in a socially disadvantaged condition.

Data about the sample are described in Table 1. No significant difference was assessed between the study and the control group at the beginning of the project.

Before the beginning of the project, the medical history of all the patients was recorded and informed consent forms for all the children/adolescents were signed by the parents/guardians.

**Figure 1:**
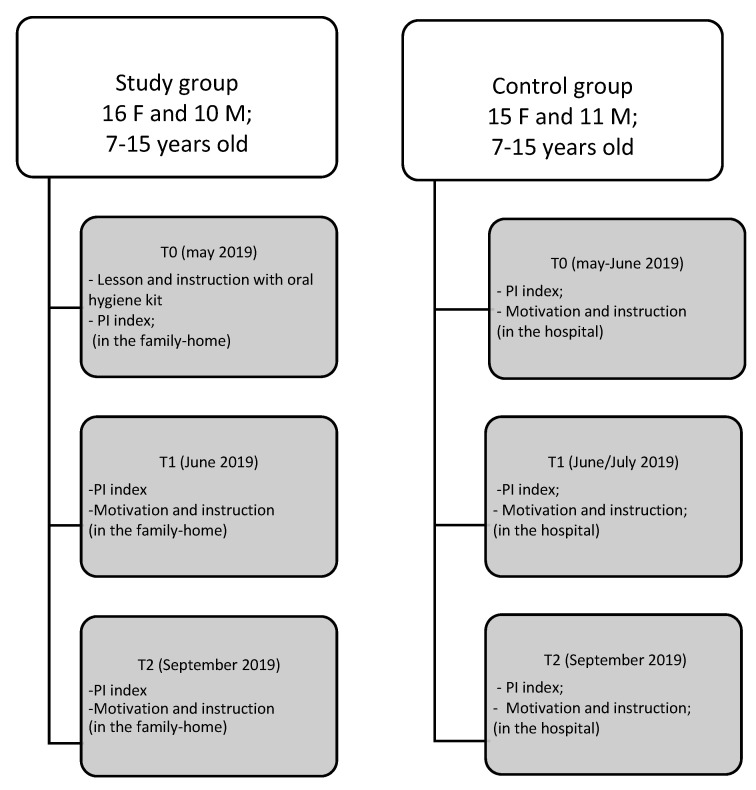
Flow chart of the study.

The flow chart of the program is described in Figure 1.

For the study group, instructions and motivation for home oral hygiene were given in the family-homes. The lesson was organized in an interactive way, with all the participants together, initially asking the participants about their knowledge on oral health and oral hygiene, and then explaining the anatomy of the tooth and the related pathologies in a simplified way. All basic oral hygiene instructions were given to all children/adolescents and their educators. Then, the collection of the plaque index (PI) was performed at T0, through the use of the plaque detector (erythrosine). The meeting was repeated after 4–7 weeks (T1), and after 10–12 weeks (T2).

For the control group, the children/adolescents were subjected to education and motivation on the dental chair, during their dental visit, through the use of the plaque detector (erythrosine), after which the PI was detected at T0.

The lesson on oral hygiene education and motivation was also addressed to their parents present in the hospital, to ensure that they received all the information on the importance of the prevention and could become a reference point for their children. The same procedure was repeated after 4–7 weeks (T1) and after 10–12 weeks (T2).

The primary outcome was the PI (O’Leary index) [15]. The O’Leary index allows the recording of bacterial plaque on the teeth surfaces, through the use of a plaque detector, allowing the parents/educators and patients themselves to notice where they had not been effective enough in the removal of plaque. Each tooth was divided into 4 sectors: mesial, distal, vestibular, and palatal/lingual. The index was calculated by dividing the number of surfaces with plaque by the total number of available surfaces.

On the basis of the initial evaluation made during the first session, it was therefore possible to prescribe to each child/adolescent the most specific aids to obtain the maximum improvement in their home oral hygiene. For example, for children with plaque in the interproximal areas between teeth, the use of dental floss was recommended; otherwise, brushing of occlusal surfaces was mostly recommended to children who showed more plaque on those surfaces.

All the children were recommended to brush their teeth a minimum of 3 times a day, for at least 2 min with a fluoride toothpaste, and were given proper instructions according to the modified Bass technique [16].

It was recommended not to put too much pressure on teeth during brushing, and to use dental floss and mouth rinse.

For the present protocol, the motivation for home oral hygiene was obtained by (a) listening to the problems perceived by the patients, encouraging their questions and being responsive to them, acknowledging their feelings, and asking them what they want to achieve (i.e., what their goals are); (b) proposing clear recommendations regarding patient-perceived problems and goals, and acknowledging that the patient does not have to accept the changes that are being recommended; (c) in a non-controlling way, explaining in terms of behavior–health contingencies why the recommendations or prescribed activities may be effective in solving perceived problems or attaining personal goals (i.e., providing meaningful rationales); and (d) encouraging patients to consider the different options and make their own choices about whether or not to endorse them [17].

### Analysis of Data

The PI values were recorded and a descriptive statistic (mean and standard deviation) was calculated. Inter-group and intra-group differences were evaluated by using parametric tests. The ANOVA test was performed to evaluate the change in each group over time. In addition, the t test for independent sample was performed to compare the study and the control groups at each stage.

Multivariate analysis of variance (MANOVA) was applied to evaluate the effect of groups and time on the plaque index.

*p*-value was set at the 0.05 level.

## 3. Results

**Figure 2:**
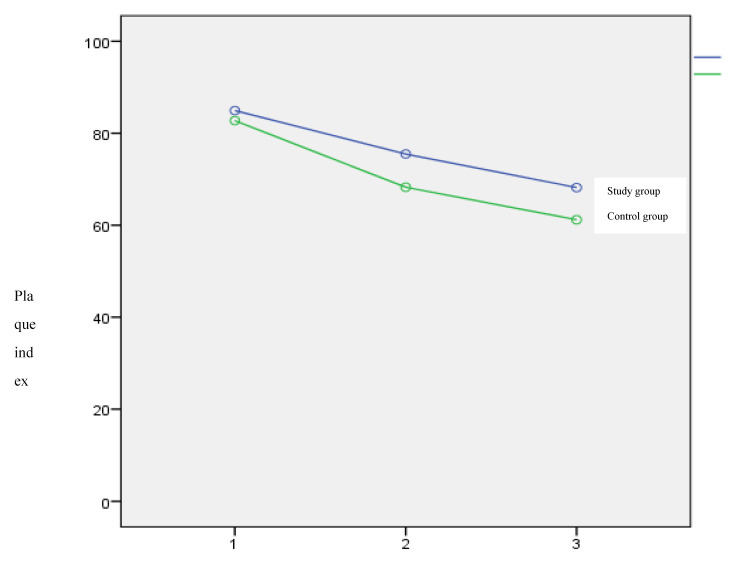
Mean value of plaque index score over time in the two groups.

The results of the PI are shown in the graph (Figure 2).

An improvement in the PI was generally found in both groups (Figure 2), with no significant differences between T0 and T1, and between T1 and T2. 

In addition, there was no statistically significant difference between the two groups all over time (Table 2).

Multivariate analysis of variance (MANOVA) revealed a statistically significant effect of time [F (1, 52) = 90.73, *p* < 0.001], from T0 to T2, regardless of the assignment group, in consequence of which the plaque index presented a moderate and significant improvement.

## 4. Discussion

This prospective controlled clinical trial was designed to evaluate the effectiveness of an oral hygiene educational and motivational program administered to a sample of children/adolescents in a disadvantaged social situation (living in a family home), and to compare the results with those of a sample matched for gender and age distribution, made up of non-disadvantaged children/adolescents.

From a social point of view, the present program was very appreciated by all participants and parents/educators. In the family-homes, the meetings created an occasion for socialization and confrontation. During the meetings, all the children/adolescents showed affection towards the operators responsible for the project, and gradually showed more and more interest and enthusiasm for all the topics and for the practical demonstrations of oral hygiene techniques. Therefore, they took home a clear message: that being able to improve oral health could have great systemic implications for the organism, for the prevention of pathologies, and therefore for the quality of life in individuals [18,19,20,21].

In particular, they were instructed on the necessity of oral hygiene, particularly during adolescence, because their microbiota is composed of cariogenic bacteria and periodontal bacteria, and are subjected to high prevalence of carious lesion [22,23,24,25].

The subjects also appeared well instructed on diet, which has been recently demonstrated to negatively influence oral health behavior and lifestyle (more snacks, less brushing, more sweets, and sweet drinks) [26,27], and about the use of chlorhexidine [28].

The results show that there were no differences between the two groups, neither at t0, nor during the study, i.e., in both groups, there was an improvement in the mean PI over time, without any significant difference between the two groups.

The present results demonstrate the efficacy of oral hygiene programs for improving the PI, without differences in the type of project. Due to the different environment in which the meetings took place (in the family-homes, for the study group, and in the dental office, for the control group), the projects were conducted with some slight differences in the protocol, which did not affect the obtained results.

The good results of prevention programs directed to disadvantaged children are also confirmed in the literature.

In 2014, a study was carried out in Pune (India) to determine the knowledge and status of oral hygiene and its change after a health education, in 100 children aged between 5 and 12 years, housed in a structure for orphans [29]. The pre and post-interventional intra-oral examination was conducted to check their oral hygiene condition. The intervention was carried out in the form of health education with demonstration of the correct brushing technique. There was a considerable improvement in the condition of their oral hygiene due to the educational intervention, and it was important for the caregivers to be instructed and trained on oral hygiene practices, so that they could supervise the children [29]. However, in that study, the results were not compared with those of a control group, and only a post-intervention examination was considered, without a T2 follow-up.

In 2017, a cross-sectional study from Turkey (Istanbul) was conducted to assess the state of oral health and oral hygiene practices of adolescents under state protection, including 55 participants aged from 12 to 18 years [30]. The plaque index was used to detect oral hygiene. The data stated the validity of their home oral hygiene practice [30]. However, that study did not include any longitudinal follow-up, nor data from a control group.

Another cross-sectional study from India (Jodhpur, Rajasthan) was conducted in 100 orphan children to assess their knowledge on oral health (their attitude toward oral hygiene and their practices) [31]. Most children revealed an adequate knowledge on their oral health, but that study lacked any control group and follow-up.

Another study, carried out in Yemen (Sana’a) [13], aimed to evaluate the prevalence of lesions of oral mucosa, caries, and oral health practices in a sample of 202 institutionalized orphaned, all males, aged from 12 to 15 years, while 202 non-orphaned students were enrolled as control subjects. Demographic data and oral hygiene practices were obtained by interviewing each subject using a special questionnaire form. The institutionalized children revealed a high prevalence of oral mucosal lesions (such as fissures in the tongue, cold sores, and traumatic ulcers), but a low prevalence of dental caries, compared with children in non-orphan schools, although they revealed poor oral hygiene practices. However, that study was based on a transversal construction, without follow-up.

In the present study, no difference was observed between the study and the control group, even at T2. This confirms that the present instructional and motivational program was perceived in the same way by children/adolescents living in family-home structures, as well as children/adolescents living with their own family, thus what seems important is the motivation to implement a habit for one’s own health, and it seems that it does not matter whether suggestions come from a parent or an educator. Recently, a systematic review underlined that a proper education about dental procedure was demonstrated to have a role in the control of pain and anxiety generally associated with dental procedure. [32]

The impact of the socio-economic level on oral health has been recently investigated and shown to have an effect mostly for pre-school children, for which the level of education of the parents can play a role [10], and also for the interceptive diagnosis of the dental problems to prevent, such as caries or malocclusion. 

## 5. Conclusions

The present data confirm the validity of educational/motivational programs to improve oral hygiene in children/adolescents, regardless of the assignment group, to obtain a significant improvement in oral health. Further investigation will aim to increase the sample size of the present pilot study.

## Figures and Tables

**Table 1:** Demographic data of the study group, with the matched control subjects.

Group	Study Group	Control Group
Total sample	26	26
Female	16	15
Males	10	11
Mean age (+/- SD)	11.71 ± 2.08	10.63 ± 1.86

**Table 2:** Mean ± SD of plaque index in the two groups and differences.

Time	Study Group	Control Group	*p*
	Mean ± SD	Mean ± SD	
T0	85 ± 19	83 ± 17	0.660 (ns)
T1	75 ± 20	68 ± 22	0.213 (ns)
T2	68 ± 18	61 ± 20	0.192 (ns)
